# Significance of chromogranin A and synaptophysin in pancreatic neuroendocrine tumors

**DOI:** 10.17305/bjbms.2020.4632

**Published:** 2020-08

**Authors:** Tatsuo Tomita

**Affiliations:** Departments of Integrative Biosciences and Pathology, Oregon Health and Science University, Portland, Oregon, USA

**Keywords:** Chromogranin A, immunohistochemistry, multiple endocrine neoplasia 1, pancreatic neuroendocrine tumors, synaptophysin

## Abstract

The two most commonly used immunohistochemical markers for neuroendocrine cells and their tumors are chromogranin A (CgA) and synaptophysin (SPY). CgA is a marker for neuroendocrine secretory granules of four pancreatic hormones and gastrin while SPY is a marker for synaptic vesicles in neuroendocrine cells, which release classic neurotransmitters such as acetylcholine and others. CgA is involved in synthesis and secretion of peptide hormones through exocytosis while the function of SPY is elusive. Thirty-five pancreatic neuroendocrine tumors (Pan-NETs) were studied, consisting of 14 insulinomas, 8 gastrinomas, 2 glucagonomas, 6 pancreatic polypeptidomas and 5 non-functioning tumors, and were immunostained for four pancreatic hormones, gastrin, CgA, and SPY. Majority of Pan-NETs were less immunostained for the endocrine hormones and CgA than the normal pancreatic endocrine cells. CgA immunostaining mostly correlates with each hormone staining in non-β-cell tumors, while SPY immunostaining recognizes endocrine cells diffusely in the cytoplasm. CgA immunostaining is less in insulinomas than in non-β-cell tumors, and CgA immunostaining may distinguish CgA-weaker insulinomas from CgA-stronger non-β-cell tumors. CgA immunostaining may be used as an independent marker for biological aggressiveness in non-β-cell Pan-NETs. The serum CgA levels are higher in subjects harboring non-β-cell tumors than those harboring insulinomas, and the serum CgA elevates in parallel to the increasing metastatic tumor mass. Thus, CgA positive immunostaining in Pan-NETs correlates with the elevated serum levels of CgA for diagnosing CgA-positive non-β-cell Pan-NETs and the increasing serum CgA levels indicate increasing metastatic tumor mass.

## INTRODUCTION

Chromogranin A (CgA) and synaptophysin (SPY) are the two most widely used immunohistochemical markers for neuroendocrine cells and their tumors, including pancreatic neuroendocrine tumors (Pan-NETs), in a pathology laboratory [1-3]. CgA is a marker for neuroendocrine secretory granules (in neurons also called large dense core vesicles) that store and release peptide hormones, originate from the trans-Golgi network, and constitute the regulated pathway of protein hormone secretion including all four pancreatic peptide hormones and gastrin [4]. CgA is an acidic protein with a molecular weight of 48 kDa consisting of 439 amino acids and is expressed by normal and tumor cells of the diffuse endocrine and neuroendocrine systems or by some cancer cells that can undergo neuroendocrine differentiation [5,6]. CgA belongs to the granin family, which includes CgA and B and secretogranins (Sg II, Sg III, Sg IV, Sg V, Sg VI, Sg VII, and Sg III)[7-9]. The endocrine secretory granules contain concentrated protein, about 0.1 g/ml including chromogranins [6]. CgA is the driving force for the biogenesis of secretory granules and induces the budding of the trans-Golgi network membranes forming dense granules [8], thus influencing the pro-hormone transport into the secretory granules [9]. To release the hormone, secretory granules make a contact with the plasma membrane (docking), and fuse with the plasma membrane (exocytosis) [6]. After cosecreting CgA with each hormone, CgA is recycled to the new cycle of hormones secretion [10,11], thus CgA modulates the endocrine secretory cycle [11-13]. SPY belongs to a family of related vesicle proteins present in small synaptic vesicle (SV), which includes synaptotagmin (p65), synaptosomal-associated protein of 25 kDa (SNAP-25), SNAP-receptor (SNARE), syntaxin, Rab3A, synaptoporin (SYNPR), pantophysin (SYPL1), mitsugumin (SYPL2), synaptogyrins 1-4 (SNG 1-4), and others [14-17]. SPY was one of the first synaptic proteins identified but its function has remained unknown to date, yet SPY appears to play a role in the SV cycle in trafficking VAP2 back to SV during endocytosis [5,16,17]. The SV is a sphere of 40 nm in diameter that stores and releases classic neurotransmitters such as acetylcholine, norepinephrine, serotonin, gamma-aminobutyric acid, glycine, histamine, and glutamate and does not contain usual secretory granules [14-17]. In pancreatic islets, secretory granules vary in sizes from 150 to 170 nm for the smallest pancreatic polypeptide (PP) cells, 150 to 220 nm for α-cells to the largest 600 nm of β-cells with a large halo inside β-granules [1,13]. Using immunoelectron microscopy with 10 nm protein A-gold complex, which we also used for growth hormone and prolactin in pituitary adenomas [18], CgA is confined to the secretory granules of islet cells revealing stronger density in α-granules than in β-granules, especially on the periphery of the granules, while SPY immunostaining is diffusely in the cytoplasm [1]. CgA is widely present in neuroendocrine cells including those of intestines, thyroid C-cells, parathyroid chief cells, anterior pituitary cells, pancreatic endocrine cells, and others [7,12]. In the endocrine pancreas, β-islet cells are weaker immunostained for CgA than non-β cells, including α-, δ- and PP cells, which are densely immunostained for CgA [1], and insulinomas show mostly lesser CgA immunostaining than non-β-cell tumors [19]. Thus, CgA immunostaining may distinguish CgA-weaker insulinomas from CgA-stronger non-β-cell Pan-NETs. CgA and SPY are colocalized in the endocrine cell cytoplasm, but CgA occurs granularly, more basically in the cytoplasm throughout gastrointestinal tract endocrine cells, corresponding to the location of neurosecretory granules, while SPY immunostaining is more diffusely outside the secretary granules, corresponding to the diffuse distribution of SV in the cytoplasm [1,6]. This report deals with differential immunohistochemical staining for CgA and SPY in several kinds of Pan-NETs aiming to reveal possibly differential immunostaining in secretory granules for CgA and SPY in cytoplasm, respectively, in different hormone-producing Pan-NETs.

## MATERIALS AND METHODS

All cases of Pan-NETs were from the University of Kansas Medical Center, Kansas City, Kansas, collected between 1975 and 2001. A total of 35 cases were included in this study, consisting of 14 insulinomas, 8 gastrinomas, 2 glucagonomas, 6 pancreatic polypeptidomas (PPomas) and 5 non-functioning Pan-NETs, the majority of which were previously reported [20,21]. All the tumors in this study were well-differentiated neuroendocrine tumors (NETs) [20] except PPoma Case 2, which was originally well-differentiated NET but was transformed to small cell carcinoma after cancer chemotherapy. The WHO classification of Pan-NETs by hormone production includes insulinoma, gastrinoma, glucagonoma, vasoactive intestinal polypeptidoma (VIPoma), somatostatinoma and non-functioning tumors; the latter include PPoma with no obvious clinical symptoms attributed to PP hypersecretion [22,23]. Therefore, clinically non-symptomatic Pan-NETs include PPomas, while clinically symptomatic Pan-NETs include insulinomas, gastrinomas, and glucagonomas in this study. Our PPoma cases were extensively studied for serum and tumor tissue PP levels [19,20,23,24]. All the tissues were routinely fixed in buffered formalin and embedded in paraffin. The archival paraffin blocks were freshly sectioned, and the paraffin sections were immunostained within 2 weeks after sectioning. The deparaffinized sections were treated with antigen retrieval procedure using citrate buffer pH 6.2. All the staining procedures were the same as previously reported immunostaining for insulin, glucagon, somatostatin, PP, and gastrin [20,21,24,25] plus monoclonal anti-CgA (Dako, Clone DAK-A3, Santa Clara, CA) and rabbit polyclonal anti-SPY (Cell Marque, Cat. 336-76, Rocklin, CA) both at 1:100 dilution. For CgA and SPY immunostaining, the normal pancreatic islets in the Pan-NET tissue sections were used as the internal controls. The immunostaining was performed with 20 sections each batch to yield good comparative staining. The clinical information on age and sex of the cases is listed (Tables 1 and 2).

**TABLE 1:**
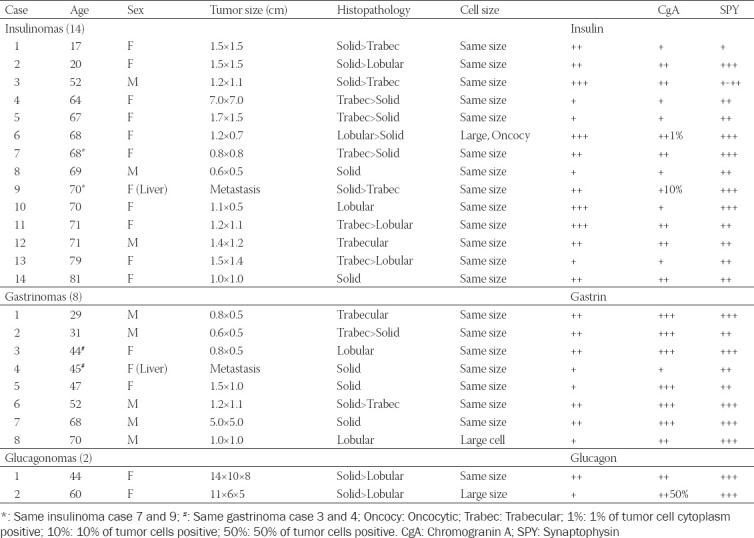
Comparative immunohistochemical staining for CgA and SPY in symptomatic pancreatic neuroendocrine tumors

**TABLE 2:**
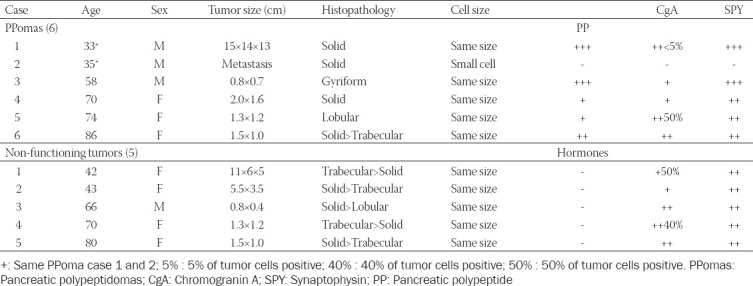
Comparative immunohistochemical staining for CgA and SPY in non-symptomatic pancreatic neuroendocrine tumors

Histopathological patterns were classified as gyriform, trabecular, lobular, solid, and anaplastic small cell [26-29]. The cytological features were divided into a) the same size of the normal islet cells, b) smaller than the normal islet cells, c) larger than the normal islet cells, and d) oxyphilic cells. For immunohistochemical staining intensity of the tumors, the staining intensity was compared to the normal islets (+++) in each Pan-NET for CgA and SPY staining, which was graded (+++) for CgA and SPY, followed by weaker staining of (++), (+), and (-), the negative staining.

## RESULTS

**FIGURE 1:**
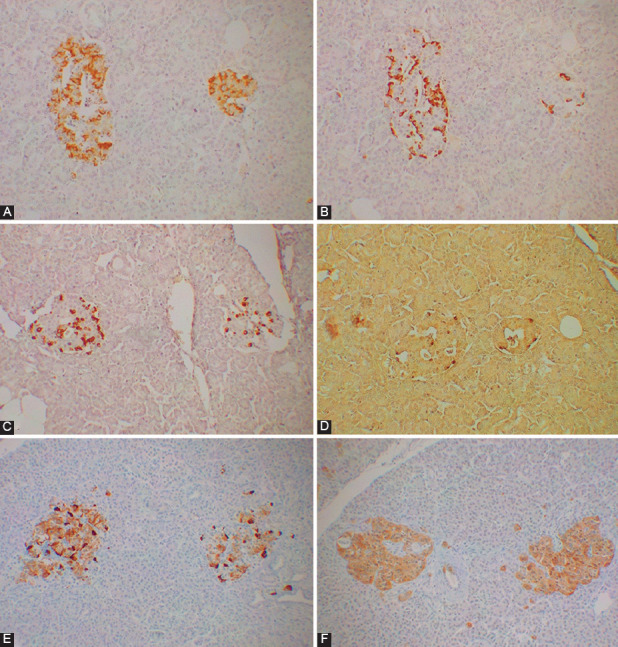
Normal islets. The major β-cells (about 70% of islet cells) contained plump cytoplasm and were strongly immunostained for insulin (A) and weak (+, right islet) to moderately (++, left islet) and granularly immunostained for chromogranin A [CgA] (E) compared to the non-β cells while the second major α-cells (about 10–20% of islet cells) contained compact cytoplasm, which were densely immunostained for glucagon (B) and CgA (+++) (E). The δ-cells (<5–10%) contained slightly plump cytoplasm (C) and slender pancreatic polypeptide (PP) cells (<1–2%) contained compact cytoplasm (D), located both within and outside the islets and both δ-cells and PP-cells were strongly immunostained for CgA (+++). All four islet cells were diffusely, moderately [left islet] (++) and strongly [right islet] (+++) immunostained for synaptophysin (SPY). A: Insulin, B: Glucagon, C: Somatostatin, D: PP, E: CgA and F: SPY immunostained.

**FIGURE 2:**
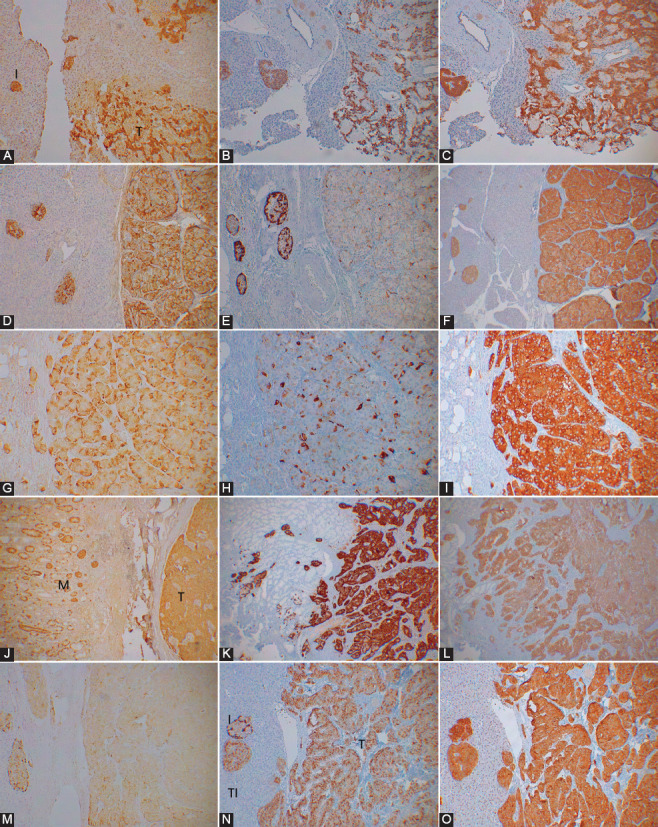
Clinically symptomatic pancreatic neuroendocrine tumors (Pan-NETs). Insulinomas, Case 3, 6 and 9. Case 3 insulinoma consisted of trabecular pattern of normal-sized, a few-cell-layered tumor cells with granularly and less insulin- and chromogranin A (CgA)-stained than normal islet cells but were strongly and diffusely stained for synaptophysin (SPY) in trabecular tumor cells (A, B and C). Case 6 consisted of lobular pattern of relatively larger oncocytic tumor cells with plump, clear cytoplasm and strong insulin staining, but less stained for CgA at 1% positive staining in the cytoplasm adjacent to the cell membrane, and were strongly and diffusely stained for SPY (D-F). Case 9 was a metastasized malignant insulinoma to the liver two years after the initial resection and consisted of mixed solid and lobular pattern of normal tumor cell size with patchy and moderately stained for insulin and sparely for CgA at 10% of tumor cells and strongly and diffusely for SPY (G, H, and I). I: Islet, T: Tumor. A, D and G: Insulin, B, E and H: CgA, C, F and I: SPY immunostained. Gastrinoma Case 3 and glucagonoma Case 2. Both normal gastrin cells and gastrinoma cells were granularly and moderately stained for gastrin and strongly stained for CgA but were diffusely and weaker stained for SPY in Case 3 gastrinoma (J, K and L). α-cells were mostly arranged at the margin of the islet lobules and lobular tumor cells were weaker stained for glucagon (M). In Case 2 glucagonoma, there were two types of islets in the adjacent pancreas: one was normal islet with normal-sized islet cells (I) and the other consisted of larger cells of the same size of the tumor cells (TI), the latter were moderately stained for CgA in 50% of tumor cell cytoplasm but strongly and diffusely stained for SPY (M, N and O). I: Islet, M: Duodenal mucosa in tissue, T: Tumor, TI: Tumor cell islet, J: Insulin. K and N: CgA, L and O: SPY, M: Glucagon immunostained.

In the normal islets, the major β-cells (about 70%) were granularly and weakly to moderately (+ to ++) immunostained in the plump cytoplasm for CgA while the second major α-cells (10–20%) were densely (+++) immunostained in the compact cytoplasm and were located at the periphery of the islet lobules (Figure 1). The δ-cells (<10%) with the slightly plump cytoplasm, located adjacent to β-cells, and slender PP cells (<5%), the fewest islet cells with the compact cytoplasm, located both within and outside the islets were also densely immunostained for CgA (Figure 1). All four islet cells were diffusely and moderately immunostained (+++) for SPY (Figure 1). Majority of PETs were mixed lobular, trabecular, and solid histopathological pattern and there were also gyriform, small cell anaplastic, and other patterns [24-27]. Majority of Pan-NETs were less or the same staining intensity of the corresponding normal pancreatic endocrine cells or gastrin cells in the duodenum due to autonomous, faster hormone secretion by the tumor cells than normal endocrine cells (Tables 1 and 2). Among 14 cases of insulinomas, 10 cases were less immunostained for insulin than normal β-cells, and four cases were as strongly immunostained for insulin of the normal β-cells (Table 1). The main histological patterns were mixed lobular and solid pattern, and some were solely lobular, solid, or trabecular pattern (Tables 1 and 2). Majority of benign insulinoma cells were of about the same size of normal islet cells with granular, less staining for insulin and CgA while SPY staining was moderately to strongly and diffusely positive in the entire cytoplasm, as seen in Case 3 (Figure 2A-C). The mostly lobular pattern, Case 6 consisted of large oncocytic cells, which were strongly and diffusely stained for insulin but patchy and linear stained for CgA at 1% of tumor cell cytoplasm adjacent to the cell membrane and strongly and diffusely immunostained for SPY (Figure 2D-F). A case of malignant insulinoma, Case 7, was mixed trabecular and solid histopathological pattern with slightly large cytoplasms, which were partly but moderately positive for insulin in all tumor cells and less stained for CgA in the tumor cell cytoplasm (about 1% of tumor cells), and were diffusely and strongly positive for SPY (Figure not shown). This case metastasized to the liver, Case 9, 2 years after enucleation, which was predominantly solid pattern and showed less insulin and moderately CgA staining in 10% of tumor cells and diffuse strong SPY staining (Figure 2G-I).

In Case 3 gastrinoma, tumor cells were granular, less stained for gastrin but strongly stained for CgA and weakly for SPY (Figure 2J-L). Case 3 was initially lobular pattern and metastasized to the liver 1 year after surgery, and the metastasized tumor was mostly solid pattern (Figure not shown).

Many α-cells were arranged along the outer margin of the normal islet lobule, and Case 2 glucagonoma cells were weaker stained for glucagon than normal α-cells but moderately stained for CgA in 50% of individual tumor cell cytoplasm and diffusely and strongly for SPY (Figure 2M-O). The adjacent pancreas showed two types of islets, namely, normal islets and neoplastic islets, and the normal islet had the same size of cytoplasm of the other normal islets with the same dense CgA and SPY immunostaining, while the neoplastic islet had larger tumor cell cytoplasm with weaker and diffusely stained for CgA and SPY (Figure 2N and O).

**FIGURE 3:**
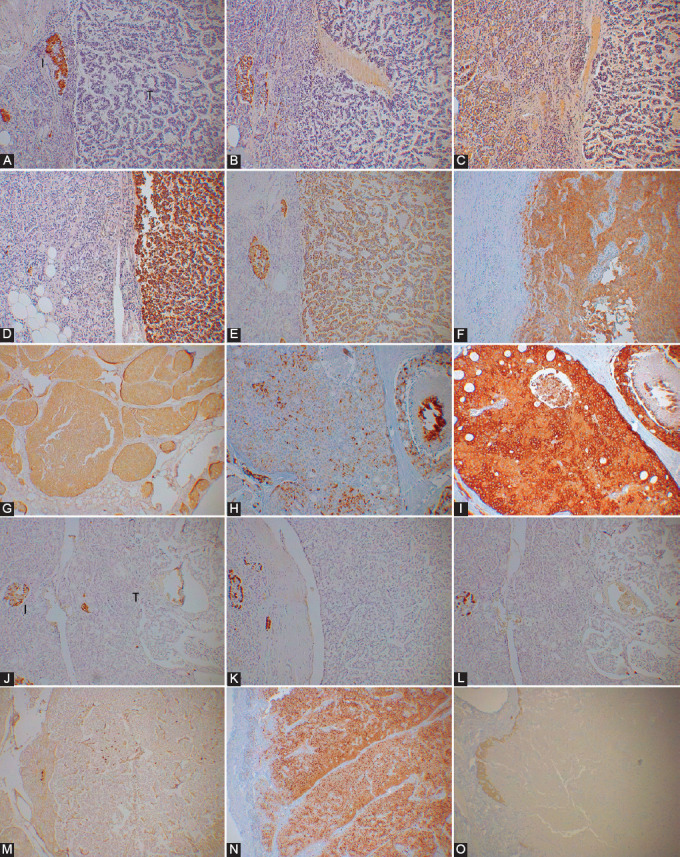
Clinically non-symptomatic pancreatic neuroendocrine tumors (Pan-NETs). PPoma Cases 3 and 1. Case 3 PPoma consisted of gyriform pattern of mostly a few-cell-layered trabecular pattern and was strongly positive for only pancreatic polypeptide (PP) and weakly and granularly stained for chromogranin A (CgA) but strongly and diffusely stained for synaptophysin [SPY] (A, B, C, D, E and F). Malignant PPoma Case 1 was a solid pattern with diffusely and moderately stained for PP and strongly stained for CgA at 5% strongly stained in tumor cells and strongly and diffusely stained for SPY (G, H and I). I: Islet, T: Tumor A: Insulin, B: Glucagon, C: Somatostatin, D and G: PP, E and H: CgA, F and I: SPY immunostained. Non-functioning Pan-NET Case 3. The tumor cells were negative for four pancreatic hormones and gastrin but moderately and diffusely positive for CgA (N) while recently infarcted mid tissue was negative for SPY staining with weaker but positive staining at the infarcted tumor margin (O). I: Islet, T: Tumor, J: Insulin, K: Glucagon, L: Somatostatin, M: PP, N: CgA, O: SPY immunostained.

A case of benign PPoma, Case 3, was gyriform pattern of a few cell-layers, which were negative for insulin, glucagon, and somatostatin but strongly positive for only PP with the same staining intensity of the normal PP cells, and tumor cells were diffusely and weakly immunostained for CgA and diffusely and strongly for SPY (Table 2, Figure 3A-F). One malignant PPoma, Case 1, from a multiple endocrine neoplasia-1 (MEN-1) family was solid pattern with moderately and granular staining for PP and moderately for CgA at 5% of tumor cell cytoplasm and diffuse strong staining for SPY (Table 2, Figure 3G-I). Case 3, non-functioning tumor, was negative for four pancreatic hormones and gastrin (Table 2, Figure 3J-M). A part of this tumor was acutely infarcted at the outer margin of the tumor with disrupted cell membrane, and the nearly entire tumor tissue remained positive for CgA while the infarcted area was negative for SPY with a weak remaining staining at the outer tumor margin, suggesting that the SV quickly disappeared after infarction while preserving CgA-positive PP secretory granules (Table 2, Figure 3N and O).

## DISCUSSION

The subjects with insulinomas and gastrinomas were diagnosed by clinical symptoms, plasma hormone levels, and radiological examination. In this study, each Pan-NET was first diagnosed for the presence of the specific hormone by immunostaining of four pancreatic hormones and gastrin, with which diagnosis of insulinoma, glucagonoma, PPoma, gastrinoma, and non-functioning tumor was rendered [20,21,25,26]. The specific hormone production also influences the prognosis of Pan-NETs since over 90% of insulinomas are reportedly benign while non-β-cell tumors, including 60–90% of gastrinomas, 50–80% of glucagonomas, over 70% of somatostatinomas, and 40–70% of vasointestinal polypeptidomas (VIPomas) are malignant [25-29] and PPomas are estimated as 60–90% malignant depending on the location and sizes of the tumors [28,29]. In clinically symptomatic insulinomas, tumors <2 cm are generally curable by surgery, while the mean size of PPoma with metastasis but no specific symptoms due to PP hypersecretion was 8.1 cm compared to 4.3 cm for those without metastasis [20]. Thus, CgA immunostaining intensity may distinguish CgA-weaker, mostly benign insulinomas from CgA-stronger, more aggressive non-β-cell tumors. Those tumors without positive immunostaining for three pancreatic hormones and gastrin were generally classified as non-functioning tumors without typical clinical symptoms of functioning Pan-NETs including PPoma; the latter does not present clinical symptoms, albeit higher serum PP levels especially after a high protein diet were reported previously by us [19,20]. In the normal pancreatic islets, β-cells were lesser immunostained for CgA than other three non-β-cells as described previously [1,13] (Figure 1) and insulinomas were relatively weaker immunostained for CgA than non-β-cell tumors but as strongly stained for SPY as in non-β-cell tumors (Table 1, Cases 7 and 9). In our 13 cases of primary insulinomas, 10 cases (Cases 1,2,3,4,5,6,8,9,10 and 13) were much weaker immunostained for CgA as reflecting the immunostaining nature of β-cell-derived tumors [1,13] (Table 1). Insulinoma Case 7 measured 0.8 × 0.8 cm and was histopathologically mixed trabecular and solid pattern, indistinguishable from the other benign insulinomas, but this tumor metastasized to the liver 3 years after the initial resection and the metastatic tumor was predominantly solid pattern (Table 1, Cases 7 and 9), corresponding to about 8% of malignancy in insulinomas, about the reported incidence of 10% malignancy in insulinomas (Table 1) [26-30]. Cases 1 and 2 insulinomas occurred in young ages, 17 and 20 years of age, respectively, and both were from the MEN-1 families (Table 1). Case 6 insulinoma consisted of oncocytic histological pattern with plump clear cells of less invasive tendency, which were linearly and patchy immunostained for CgA in 1% of tumor cell cytoplasm arranged parallel to the cell membrane, probably pushed by numerous mitochondria in the cytoplasm but diffusely stained for SPY (Figure 1D-F). Our Pan-NETs cases were diagnosed clinically and resected by our surgeon at the University of Kansas Medical Center, the late Dr Stan Friesen, who screened for serum PP levels after a high-protein meal among the family members of MEN-1 [19,20], yielding a higher percentage of gastrinoma and PPoma cases than the other studies. Subjects with MEN-1 syndrome were reported to develop Pan-NETs in 60–70% of the cases and gastrinomas are the most common Pan-NET, occurring at 40% of cases in the gastrinoma triangle (superiorly in the junction of cystic duct and common bile duct, inferiorly in the junction of the second and third portion of the duodenum, and medially in the junction of the neck and body of the pancreas)[31-33], 60% in duodenum and 30% in the pancreatic head [32]. Duodenal gastrinomas are usually small and multiple (<1 cm in 77%, mean 0.9 cm), which follow a good prognosis after resection, while pancreatic gastrinomas are generally larger (<1 cm 6%, mean 3.8 cm) and follow a worse prognosis [31,32]. There have been quite different Pan-NET statistics in the MEN-1 cases including the two well-cited reports [33,34]. Among 130 MEN-1 cases admitted to the National Institutes of Health Hospital, 86 cases (66%) were found to have Pan-NETs, in which 61 cases (47%) were gastrinomas, 15 cases (12%) insulinomas, and 5 cases (4%) non-functioning Pan-NETs [32]. A later study from the European hospital reported that 70% of MEN-1 subjects had Pan-NETs including 40% of gastrinomas, 10% of insulinomas, and 20% of non-functioning Pan-NETs [33]. More detailed studies were also reported. In a study of 28 subjects with MEN-1, 100 PETs were detected, among which 77 tumors were positive for the following hormones: 37 - glucagon, 27 - insulin, 11 - PP, 1 - gastrin, 1 - VIP, and 7 - unclassified, however this study represents unusual statistics compared to the other studies [35]. A multi-institutional study revealed Pan-NETs detected in 80–100% of the MEN-1 subjects, in whom clinically non-symptomatic tumors - 100%, gastrinomas - 54%, insulinomas - 21%, and glucagonomas - 3% despite being non-functioning state albeit positive immunostaining for the hormones in the early stage of Pan-NETs [32]. In a Swedish institute, where 324 cases of Pan-NETs were studied, non-functioning tumors were the most common at 59%, followed by insulinomas (17%), gastrinomas (13%), and VIPomas (5%) [35]. Gastrinomas are potentially invasive and fatal tumors like other non-β-cell tumors, which metastasize to the liver at 60–90% [36-38] except small tumors in the duodenal submucosa (Figure 2J-L, Table 1), which clinically present an early peptic ulcer syndrome, Zollinger–Ellison syndrome and follow a better prognosis after resection than the same tumor in the pancreatic head [20,38,39]. Gastrinoma cells were strongly stained for SPY, suggesting active SV involved in possible autonomous gastrin secretion through endocytosis [12]. We found a disproportionally higher PPoma cases in our study by detecting high serum PP levels by radioimmunoassay after a high-protein meal, since we performed immunostaining for PP as well as PP tissue levels, and we believe that the real incidence of PPomas may be much higher than reported in the literature, since not all Pan-NETs are routinely studied for PP in a regular pathology laboratory especially those with no specific clinical symptomatic Pan-NETs [20,21,40,41]. Indeed, Burke et al. reported that the most common Pan-NET in MEN-1 patients is functioning Pan-NET including gastrinoma [36]. Our non-symptomatic cases included a total of 11 cases at 31% among 35 cases, consisting of 6 PPomas and 5 hormone-negative tumors, and non-symptomatic cases were second common after insulinoma of 14 cases [40%] (Tables 1 and 2). Case 1 PPoma was a huge tumor occupying the bulk of body and tail of the pancreas, 15 × 14 × 13 cm, and solid pattern of the histopathology was probable malignant Pan-NET. The tumor metastasized to the liver after hemipancreatectomy and spread diffusely to the remaining pancreas, liver, lungs, and bone marrows after chemotherapy, two and a half years later the histopathology of the recurrent tumor was small cell anaplastic tumor, which was negative to PP, CgA, and SPY (Table 1, Figure not shown)[20,21]. Our Pan-NETs were well-differentiated [21], and CgA immunostaining should be compared among the Pan-NETs of the same differentiation since less differentiated PET may not show strong CgA staining than well-differentiated ones such as CgA-negative small cell carcinoma (Table 2). The presence of CgA and SPY in the non-functioning tumors may represent mutated, inactive hormone secretory granules undetectable by specific anti-hormone antibodies or unknown hormones inside the secretory granules.

Glucose-induced insulin secretion consists of typical two phases of insulin secretion in both *in vivo* and *in vitro*: an early small peak before glucose is metabolized within 5 min exposed to a high-glucose and the larger second phase secretion is after 20–30 min glucose infusion mediated through glucose metabolism [42]. The early phase of insulin secretion is similar in neurotransmitter secretion at the nerve ending through SV without obvious secretory granules [1,2,5]. The SV of the readily releasable pool in the synapses is docked to the cell membrane and release neurotransmitters from the SV through endocytosis on stimulation in a similar mode of secretory granules secretion [5,9]. It has been suggested that neuroendocrine cells including pancreatic islet cells may secrete peptide hormone mostly through exocytosis of secretory granules fusing with the cell membrane, which represent the second phase of insulin secretion, while the early spike of insulin secretion may be secreted through SV endocytosis since neuroendocrine cells are equipped with both secretory granules for exocytosis in a typical peptide hormone secretory mechanism and also with SV through endocytosis, the latter is the main secretory system for neurotransmitter, which takes place instantaneously in a matter of split seconds [5]. This early phase of glucose-induced insulin secretion is modulated through glucose receptor before glucose is metabolized and is thought to be mediated via glucose-kinase in the β-islet cells [43,44]. The stronger staining of SPY than CgA in insulinomas may also implicate robust SPY participation in insulin secretion through endocytosis. The other functioning Pan-NETs including gastrinomas and glucagonomas are also more strongly positive for SPY than CgA, suggesting active SV involvement on the early gastrin and glucagon section, respectively.

In non-β-cell Pan-NETs, hormone immunostaining mostly correlates with that of CgA immunostaining, supporting that each hormone synthesis parallels with CgA synthesis, while SPY immunostaining is quite different from the hormone and CgA immunostaining and this may support two secretory mechanisms in normal islet cells and Pan-NETs: one through CgA in exocytosis and another through SV in endocytosis. In our cases, those with moderate CgA immunostaining (>++) in mixed more solid and less trabecular or lobular pattern may be considered as potentially malignant, which are more common in non-β-cell tumors than in insulinomas (Tables 1 and 2). Serum levels of CgA, neuron specific enolase, and α-subunit of glycoprotein hormones were elevated in 50%, 43%, and 24% of patients with NETs, respectively [45]. Markedly elevated serum CgA levels, more than 300 ng/ml, were observed in only 2% of control patients compared to 40% of patients with NETs [45]. Thus, serum CgA levels are most specific among three markers, CgA, neuron specific enolase, and α-subunit of glycoprotein hormones in patients with NETs [45]. The baseline serum CgA levels were elevated in 103 of 208 patients (50%) with various NETs, including carcinoid tumors, insulinomas, gastrinomas, non-functioning Pan-NETs, pheochromocytomas, medullary thyroid tumors, neuroblastomas, Merkel cell tumors, and pituitary adenomas [44]. However, the elevated serum CgA was rarely present in subjects with pituitary adenomas (13%), insulinomas (10%), and paragangliomas (8%) [45]. The baseline serum CgA and PP were about the same at 100–150 ng/ml, and elevated 30–90 min after a meal and reached 2–3 times above the baseline levels [20,40,44,45], and post-protein-meal serum CgA would be much higher in subjects with NETs [21,39]. Thus, the combined post-protein-meal serum CgA and PP measurement will increase the early detection of gastroenteropancreatic NETs (GEP-NETs) [45-48]. Elevated serum CgA levels were reported in 100% of gastrinomas, 89% of pheochromocytomas, 80% of carcinoids, 50% of medullary thyroid carcinomas, and in 69% of non-functioning Pan-NETs, respectively [44,48,49]. Subjects with both functioning and non-functioning Pan-NETs showed up to 60–80 times higher serum CgA levels of the upper reference range [45,46,49]. The mean serum CgA levels in the subjects with carcinoid tumors, insulinomas, gastrinomas, and non-functioning Pan-NETs were 688 ng/ml, 105 ng/ml, 772 ng/ml and 306 ng/ml, respectively, as compared to the control levels of about 100 ng/ml [45]. The maximal serum CgA levels were reported in patients with carcinoid tumors, insulinomas, gastrinomas, and non-functioning Pan-NETs at 5200 ng/ml, 236 ng/ml, 1900 ng/ml, and 14,700 ng/ml, respectively [45]. There was also a correlation between serum CgA levels and tumor progression: elevated serum CgA levels were reported in 83% of GEP-NETs and elevated serum CgA levels were present in 100% of cases with liver metastasis [49,50]. In GEP-NETs, high serum CgA levels correlate with shorter survival and liver metastasis as reported in small intestinal NETs with up to 200 times above normal levels and in MEN-1 cases up to 150 times higher levels [49-51]. Furthermore, a sudden increase in serum CgA was accompanied by rapid tumor growth and short survival [52]. In Pan-NETs, both functioning and non-functioning Pan-NETs showed serum CgA levels up to 60–80 times the upper normal levels, particularly in Zollinger–Ellison syndrome in MEN-1 cases with serum CgA levels being 80–100 time higher than the upper normal levels [45,48]. So far, serum CgA levels are widely accepted as the marker for GEP-NETs [45,46,52]. This study may support a good correlation between CgA immunohistochemical staining and serum CgA levels in Pan-NETs where the strong CgA immunohistochemical staining appears to coincide with higher serum levels. A corroborative study between CgA immunohistochemistry of Pan-NET tissue and serum CgA levels has not been reported to date and such study is warranted. In insulinomas, which contain less CgA than the other non-β-cell tumors, serum CgA levels are not increased in the patients but measurement of serum CgA is a helpful indicator for tumor metastasis by the increasing CgA-secreting tumor mass [53-55].

Thus, simple and reliable CgA study may be used for an indirect, independent diagnostic and prognostic marker in GEP-NETs in three folds: first to distinguish more benign insulinomas from more aggressive non-β-cell tumors; second, to access the degree of malignancy for primary non-β-cell tumors by the CgA staining intensity; and third, increasing serum CgA levels as an indicator of growing and metastatic tumors, since elevated serum CgA levels suggest growing tumor sizes and metastatic tumors.

The disappearance of SPY immunostaining from the acute infarcted area of a PET further supports the quick turnover of SV while still preserving secretory granules, as seen in the immunostained CgA in the infarcted Pan-NET cell cytoplasm (Figure 3N and O) [56].

A further study for comparative CgA levels of tumor tissue and serum CgA levels in Pan-NETs is warranted to prove possible feasibility of CgA immunostaining to distinguish benign Pan-NETs and other NETs from malignant counterparts for initial diagnosis and clinical follow-ups.
